# Aging-dependent immunological changes in multiple sclerosis

**DOI:** 10.3389/fimmu.2025.1663526

**Published:** 2025-10-02

**Authors:** Andrea Iribarren-López, Laura Martins-Almeida, Jadon K Wells, Tamara Castillo-Triviño, Álvaro Prada, Hilda A Pickett, Ainhoa Alberro, David Otaegui

**Affiliations:** ^1^ Neuroimmunology Group, Neurosciences Area, Biogipuzkoa Health Research Institute, San Sebastian, Spain; ^2^ Centro de Investigación Biomédica en Red de Enfermedades Neurodegenerativas, Instituto de Salud Carlos III, Madrid, Spain; ^3^ Telomere Length Regulation Unit, Children’s Medical Research Institute, University of Sydney, Westmead, NSW, Australia; ^4^ Neurology Department, Donostia University Hospital, San Sebastian, Spain; ^5^ UGC Laboratories Gipuzkoa, Immunology Department, Osakidetza Basque Health Service, San Sebastian, Spain

**Keywords:** multiple sclerosis, immunosenescence, aging, neuroimmunology, inflammaging, cell senescence, thymic involution, telomere attrition

## Abstract

Multiple sclerosis (MS) is a chronic inflammatory and neurodegenerative disease in which immune dysregulation plays a central role. As the life expectancy of people with MS (pwMS) increases, understanding how aging affects their immune system—collectively referred to as immunosenescence—has become crucial. In this study, we characterized immunosenescence in pwMS by analyzing age-related changes in the immune system. To fulfill this, blood samples were collected from pwMS and healthy controls (HCs) of independent cohorts: i) immune cell populations were assessed in PBMCs (n= 110), ii) thymic involution and telomere attrition were measured in DNA samples (n=150), and iii) inflammatory and neurodegeneration markers were evaluated in plasma (n=146).Our results revealed distinct age-associated alterations in immune cell subsets between pwMS and HCs, including B and NK cells. Notably, pwMS showed an age-related increase in CD28–CD57+ and CD28+CD57+ cells in CD4+ and CD8+ T cells. Thymic involution was reported with age in both groups and, importantly, we found a more pronounced thymic involution in younger pwMS. A positive correlation was found between age and the levels of IL-6, TNF-α, and CRP in pwMS, results consistent with the inflammaging phenomenon. Similarly, NFL levels were elevated in pwMS and correlated positively with age in both groups., Remarkably, we found a positive correlation between NFL levels and IL-6, and between NFL levels and TNF-α only in pwMS. Telomere shortening occurred with age in both groups, without significant differences. Notably, our study provides an integrative and multi-biomarker characterization of immune aging process in pwMS, revealing new insights into this complex relationship. These findings highlight specific age-related immune alterations in MS and underscore the importance of incorporating age and immunosenescence monitoring into MS clinical management and therapeutic strategies.

## 1 Introduction

Multiple sclerosis (MS) is a chronic, inflammatory, and neurodegenerative disease mediated by autoimmune reactions that cause the destruction of myelin in the central nervous system (CNS) (1). MS is characterized by immune dysregulation, which leads to demyelination, axonal damage, and neurodegeneration, resulting in neurological and physical disability (1, 2). MS is a heterogeneous and multifactorial disease influenced by both genetic and environmental factors (3, 4).

Although the exact etiology of the disease remains unclear, it is known that it is based on a dysregulation of the immune system, which involves both the innate and adaptive immune systems (5–8). The most accepted hypothesis to explain the immunopathogenesis of MS involves an initial activation of T cells reactive to myelin antigens in peripheral lymph tissues. Activated autoreactive T cells subsequently infiltrate the CNS, where they are reactivated by the recognition of myelin by antigen presenting cells in the CNS, initiating demyelination and tissue damage. These immune events provoke the activation of astrocytes and microglia, oligodendrocyte death and, potentially, axonal loss. In addition, the release of proinflammatory mediators facilitates the permeabilization of the blood-brain barrier (BBB), attracting monocytes and additional lymphocytes to the CNS (8).

Regarding immune cell dysregulation, CD4+ cell differentiation into proinflammatory Th1 and Th17 lymphocytes has been described (1, 9, 10). Also contributing to the dysregulation of the T-cell response, there is a decrease in the number and/or activity of regulatory T cells (1, 11, 12). Similarly, NK cells are reduced in people with MS (pwMS) and appear to play an immunoregulatory role in the disease (13). Further contributions to CNS damage in MS are associated with B cell activation, antigen presentation function and antibody production (1, 7).

Unlike other neurodegenerative conditions, the global average age of MS diagnosis is 32 years, which means that people live with the disease for decades (14). Indeed, recent data indicate that almost 25% of pwMS worldwide are over 60 years (15). These data confirm the aging of the MS population, probably due to the increasing longevity of the general population and the availability of effective disease-modifying treatments (16). Therefore, there is an overlap of the typical processes and pathologies of aging with those of MS, which brings the impact of aging on disease progression to the forefront.

Aging markedly affects the immune system, as it is associated with a continuous decline in immune integrity and efficiency, termed immunosenescence (17, 18). Immunosenescence is characterized by altered immune cell phenotype and response, thymic involution, telomere attrition, oxidative stress, and a chronic low-grade inflammation, among others, leading to a poor immune response (19–22).

Considering the impact of age on MS disease and on the immune system, it is crucial to understand how this biological process influences the immune system of pwMS. Previous works found aging-associated biomarkers during the course of MS, suggesting ongoing immunosenescence in the disease, and even premature immunosenescence in the case of some biomarkers (8, 16, 18, 23, 24). These age-associated biomarkers include: thymic involution, reduced CD4+/CD8+ ratio, increased memory T cells, loss of CD28 antigen, expansion of age-associated B cells (with proinflammatory characteristics), reduction of telomere length, and increased oxidative stress (24–31). In addition, Dema et al. recently publish a complete work about the role of immunosenescence in the mouse model of MS.

However, a comprehensive understanding of the association between immunosenescence and MS remains elusive in humans. To address this, our study provides an exhaustive characterization of immunosenescence in pwMS in a cohort with a wide age range. We performed a detailed analysis encompassing immune cell populations, thymic involution, inflammatory mediators, neurodegeneration biomarkers, and telomere attrition. This in-depth approach will helps to gain further insight into the interplay between aging and MS pathophysiology.

## 2 Materials and methods

### 2.1 Participants

The study has been conducted at the Neuroimmunology group of Biogipuzkoa Health Research Institute (Biogipuzkoa HRI) and samples were collected in collaboration with the Neurology and Immunology departments of the Donostia University Hospital (HUD) and the Basque Biobank (www.biobancovasco.org). The CEIm-E Ethics Committee approved the study (PI2020075 and PI+CES-BIOEF 2023-08) and all donors provided written informed consent before blood sampling.

Three independent cohorts were analyzed: a peripheral blood mononuclear cells (PBMCs) cohort (n=110), a DNA cohort (n=150), and a plasma cohort (n=146). Each cohort included healthy controls (HC) and pwMS. To obtain a whole picture, no exclusion criteria was set based on EDSS score, MS subtype and treatment. Information for each cohort is presented in **Table 1**. The PBMCs, DNA and Plasma cohorts were selected to include samples from a broad age range (individuals under 20, 20-29, 30-39, 40-49, 50-59, 60-69, 70-79, and over 80 years old). Detailed information about the age ranges of each cohort is provided in **Supplementary Table S1**.

**Table 1:** Cohorts included in the study.

Cohort	Group	Sex	Age (years old)		
			Mean age	SD	Min-Max
PBMCs Cohort (n=110)	HC (n=42)	Female 80.9%	35.2	± 13.0	22-60
		Male 19.1%	36.0	± 12.4	24-64
	pwMS (n=68)	Female 76.4%	44.5	± 10.7	18-82
		Male 23.6%	46.0	± 2.4	30-69
DNA Cohort (n=150)	HC (n=75)	Female 52,6%	55,4	± 24.5	13-100
		Male 41.3%	49.7	± 23.7	19-94
	pwMS (n=75)	Female 56.0%	50.3	± 20.0	16-82
		Male 44.0%	45.1	± 19.0	15-78
Plasma Cohort (n=142)	HC (n=66)	Female 50.0%	52.6	± 19.6	21-87
		Male 50.0%	52.2	± 19.7	21-90
	pwMS (n=76)	Female 52.6%	49.2	± 23.0	13-80
		Male 47.4%	45.0	± 20.9	15-78

### 2.2 Sample extraction

Whole blood samples from all individuals were obtained by venipuncture in EDTA and sodium heparin tubes (Vacutainer, BD Biosciences) and processed within 1 hour of sampling. PBMCs were isolated by density gradient centrifugation with Lymphoprep™ (Stemcell), following manufacturer’s instructions. Cells were frozen in RPMI 1640 Medium (Gibco, ThermoFisher) with DMSO and stored in liquid nitrogen. EDTA tubes were centrifuged at 1258g for 20 min to recover plasma. Plasma samples were aliquoted and stored at − 80 °C. Then, DNA from leukocytes was extracted with the FlexiGene DNA Kit (Qiagen), aliquoted and stored at − 80 °C.

### 2.3 PBMC culture and flow cytometry

For the characterization of PBMCs three different multicolor flow cytometry (FC) panels were designed and acquired in a SH800 Cell Sorter (Sony). Panel 1 was designed for the characterization of the main immunological populations (T lymphocytes, B lymphocytes, NK cells and monocytes), Panel 2 for T cell subtypes as well as markers of aging, and Panel 3 for the immunological response. All the cell populations and the markers used to classify them are summarized in **Table 2**. To simplify the presentation of our flow cytometry data, we have adapted the nomenclature for T cell subpopulations based on CD28 and CD57 expression as described by Pangrazzi et al. (32).

**Table 2:** List of immune cell populations analyzed by flow cytometry and their corresponding markers.

Immune cell population subsets	Markers
Panel 1	
Alive cells	7AAD-
Lymphocytes	
T lymphocytes	CD3+
B lymphocytes	CD19+
Natural Killer cells	CD56+
Monocytes	
Classical monocytes	CD14++ CD16-
Intermediate monocytes	CD14+ CD16+
Non-classical monocytes	CD14+ CD16++
Panel 2	
Alive cells	7AAD-
T lymphocytes	CD3+
T helper lymphocytes	CD4+
T cytotoxic lymphocytes	CD8+
Aging markers	
Activation capacity	CD28+
Senescence	CD57+
Aging subsets	
Early activated T lymphocytes	CD28+CD57-
Activated T lymphocytes	CD28+CD57+
Early senescent T lymphocytes	CD28-CD57-
Senescent T lymphocytes	CD28-CD57+
Panel 3	
Alive cells	7AAD-
Lymphocytes	
T helper lymphocytes	CD4+
Active T helper lymphocytes	CD69+
Th17 lymphocytes	CD196+
T regulatory lymphocytes	CD25+CD127-

Note that they have been classified by subfamilies and the general marker for the family (such as the CD3+ for all the T lymphocytes) has been omitted for more clarity.

For the FC analysis, PBMC samples were thawed washed to remove possible traces of DMSO and the number of cells was counted. From each sample, we prepared 6 aliquots of 100,000 cells in 100 µl. Four of these tubes were immediately processed for FC (Panel 1 and Panel 2). The other 2 aliquots were cultured in 96-well plates (100,000 cells in 100 µl of RPMI medium in each well) and incubated for 24 hours with PHA (1 µg for 100 µl of medium) to stimulate the immune response. Then, PBMCs were collected and processed for FC (Panel 3).

Samples were washed and blocked with DPBS with BSA at 5% (1.1 mL). Next, tubes were centrifuged for 5 minutes at 400 g and 1 mL of supernatant was removed. The remaining 200 µl were resuspended and incubated with the antibodies shown in **Table 2** for 20 minutes in the dark at room temperature. Subsequently, the samples were washed with 1 mL of DPBS-BSA 5% and then centrifuged for 5 minutes at 400 g. 1 mL of supernatant was removed and the remaining 200 µl were resuspended and taken to the flow cytometer for analysis.

The software of the Sony SH800 Cell Sorter was used to establish the gates and the compensation matrix. The obtained percentage data were extracted from this software and analyzed as described in the statistics section below.

### 2.4 Quantitative PCR

For the signal-joint T cell receptor (TCR) excision circles (sjTREC) analysis, qPCR was performed using the CFX384 Touch™ Real-Time PCR Detection System and the data were analyzed using CFX Maestro™ Software. The protocol was based on the work of Ou, X. L. et al. (33) and several changes were made during the set-up.

For the sjTREC reaction, the PCR mixture was: 5 µl of TaqMan Premix (TaqMan™ Genotyping Master Mix, 4371355, Applied Biosystems™), 250 nM of each set of primers, 250 nM of the TaqMan hydrolysis probe, 2 µl of DNA (100 ng/µl DNA) and H_2_0 up to 10 µl of final volume. The sequence of the primers and the probe for sjTREC were: 5′-CCATGCTGACACCTCTGGTT-3′ (Forward primer), 5′-TCGTGAGAACGGTGAATGAAG-3′ (Reverse primer), and 5′-FAM-CACGGTGATGCATAG GCACCTGC-TAMRA-3′ (TaqMan probe).

For the endogenous reaction, the TaqMan™ Copy Number Reference Assay, human, RNase P kit (4403326, Applied Biosystems™) was used, which measures RPPH1. This gene codes for the H1 component of the H1 RNA P ribonuclease (H1RNA) and is used as a standard reference assay for copy number analysis. The RNAse P PCR mixture was: 5 µl of TaqMan Premix (TaqMan™ Genotyping Master Mix, 4371355, Applied Biosystems™), 0.5 of the VIC™ dye–labeled TAMRA™ probe, 2 µl of DNA (100 ng/µl DNA) and H20 up to 10 µl of final volume.

Thermal cycling conditions were 95°C for 10 minutes, followed by 50 cycles of 95°C for 15 seconds and 60°C (annealing temperature for sjTREC) for 60 seconds. Then, a dissociation curve was performed to ensure the presence of a single PCR product.

### 2.5 Immunoassays for inflammatory and neurodegenerative Markers

Different molecules were measured in plasma using the Ella™ (ProteinSimple, bio-techne) automated enzyme-linked immunosorbent (ELISA) platform. The cytokines IL-10, IL-1β, IL-6, IL-8 and TNF-α were measured using a custom multi-analyte cartridge designed for this assay (#SPCK-PANEL, bio-techne). Following manufacturer’s instructions, plasma samples were diluted 1:2 and loaded in the cartridge. C-Reactive Protein (CRP) was analyzed in an individual cartridge (SPCKB-PS-000200, bio-techne). For this cartridge, plasma samples were prepared at a 1:2,000 dilution.

As a neurodegeneration biomarker, neurofilament light (NFL) was measured in plasma samples. For this analysis, Simple Plex Human NF-L Cartridge (SPCKB-PS-002448, bio-techne) was used. Plasma samples were prepared at a 1:2 dilution. The results obtained were analyzed as described in the statistics section.

### 2.6 Determination of telomere length by TRF analysis

Terminal Restriction Fragment (TRF) length analysis was performed as previously described by Lu. et al. (34), with small modifications. Briefly, genomic DNA was digested to completion with 50U each of the restriction enzymes HinfI and RsaI (New England BioLabs) by incubation at 37°C overnight, followed by heat denaturation at 80°C for 10 minutes. Digested DNA was separated by pulsed-field gel electrophoresis using the *CHEF-DR II system* (Bio-Rad), stained with ethidium bromide, and then imaged under UV (UVP Gel Solo) to confirm DNA digestion.

Agarose gels were then vacuum-dried at 80°C for 45 minutes, incubated in denaturation solution (0.5 N NaOH and 1.5 M NaCl) for 1 h with gentle rocking, followed by incubation in neutralization solution (0.7 M Tris-HCl, 1.5 M NaCl, pH 8) for 1 h with gentle rocking. Gels were briefly washed in 2x SSC before hybridization with γ-[^32^P]-ATP-labeled (GGGTTA)_4_ in hybridization solution (Merck) at 50°C with end over end rotation, overnight.

After hybridization, gels were washed, and exposed to a Phosphor Imager screen (Cytiva) for 48 hours. Phosphor screens were scanned using a Typhoon biomolecular imager (Cytiva) at 4000 intensity (AU). After imaging, telomere length was analyzed using *ImageQuant software* to provide an estimation of the mean telomere length of the sample.

### 2.7 Statistics

*IBM SPSS version 23* and *R-Studio* with *R version 4.1.2 statistical software* were used for the statistical analyses. Shapiro-Wilk was used as normality test. T-student and Mann-Whitney-Wilcoxon tests were used for mean comparisons between groups for normally and non-normally distributed variables correspondingly. Pearson and Spearman correlation tests were conducted to assess the correlations in normally and non-normally distributed variables respectively. The results were plotted with *GraphPad Prism version 8.0.1*. For all the analyses significance was set at p-value<0.05 and presented as *p<0.05; **p<0.01; ***p<0.001; ****p<0.0001.

## 3 Results

### 3.1 Immunological changes between people with MS and healthy controls

In the flow cytometry experiments of the PBMC cohort, 29 immune cell populations were analyzed – identified with the markers presented in **Table 2**. Comparison of these 29 immune populations was made between the two study groups, and 8 populations showed significant differences between pwMS and HCs (**Table 3**).

**Table 3:** Immune cell populations found to be different in people with MS and healthy controls.

Immune cell populations	Mean percentage (SD) in HC	Mean percentage (SD) in pwMS	p-value
CD28+ T lymphocytes CD3+ CD28+	81.20 (± 7.56)	83.15 (± 8.93)	0.046
Activated T lymphocytes CD3 + CD28+ CD57+	5.40 (± 4.31)	6.51 (± 3.74)	0.036
Early senescent T lymphocytes CD3 + CD28- CD57-	19.89 (± 7.53)	16.33 (± 6.06)	0.007
T helper lymphocytes CD3+ CD4+	79.74 (± 7.75)	82.43 (± 9.29)	0.032
Early senescent Th lymphocytes CD3+ CD4+ CD28- CD57-	14.46 (± 6.61)	11.75 (± 6.45)	0.038
CD28+ Tc lymphocytes CD3+ CD8+ CD28+	17.40 (± 7.40)	22.30 (± 10.05)	0.003
Early activated Tc lymphocytes CD3+ CD8+ CD28 + CD57-	4.91 (± 2.32)	7.43 (± 4.79)	0.001
Activated Tc lymphocytes CD3+ CD8+ CD28+CD57+	0.19 (± 0.22)	0.33 (± 0.38)	0.043

Mean percentage and standard deviation results are shown for each of the populations and groups.

Significant differences were observed in T helper lymphocytes, which were significantly increased in pwMS (p=0.032). All the other main immune cell populations (B cells, NK cells and monocytes) did not show significant differences in pwMS.

The rest of the significant differences are in T cell subpopulations identified based on CD28 and CD57 expression, the markers used to study senescence. The subpopulations expressing CD28 are found to be significantly increased in pwMS: CD28+ T cells (p=0.046), CD28+ Tc cells (p=0.003), early activated Tc cells (p=0.001) and activated Tc lymphocytes (p=0.043). In contrast, populations that do not express CD28 antigen are significantly decreased in the pwMS group; early senescent T cells (p=0.007) and early senescent Th cells (p=0.038) (**Table 3**).

### 3.2 Age-related correlations of immune cell populations differ between people with MS and healthy controls

**Figure 1:**
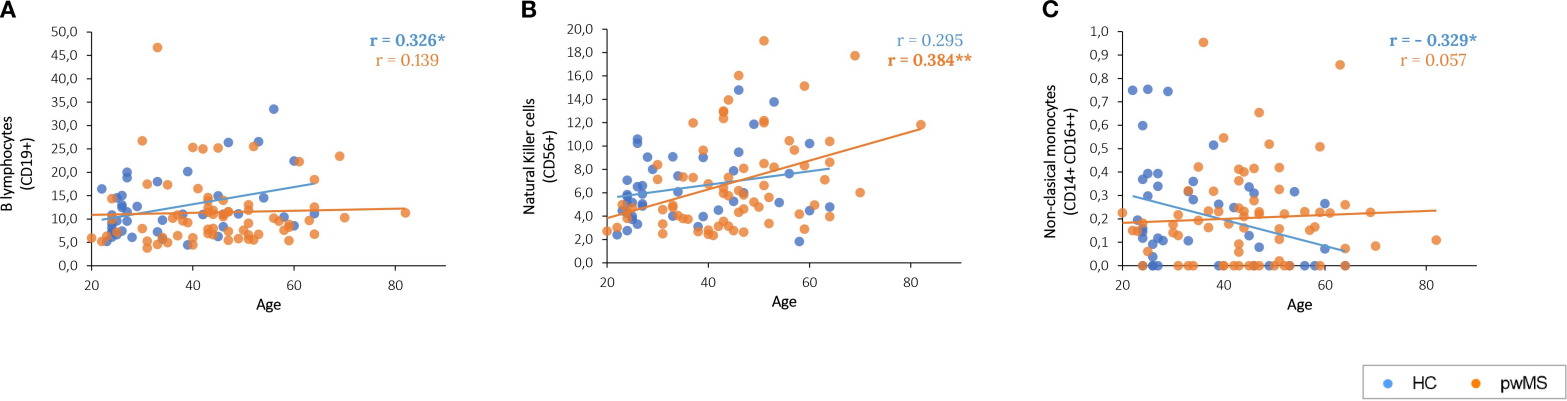
Graphical representation of the significant correlations between age and the main immune populations. **(A)** B lymphocytes correlation with age. **(B)** Age-related correlations of Natural Killer cells. **(C)** Age-related correlations of cytotoxic non-classical monocytes.

Age correlation analysis was performed for each of the groups. Of the 29 cell populations analyzed, 12 presented a significant correlation with age in at least one of the groups (**Supplementary Table S2**). The main immune cell populations results showed that B cell proportions increase and non-classical monocytes decrease with age only in HCs. Moreover, NK lymphocytes increase with age in pwMS (**Supplementary Table S2** and **Figure 1**).

**Figure 2:**
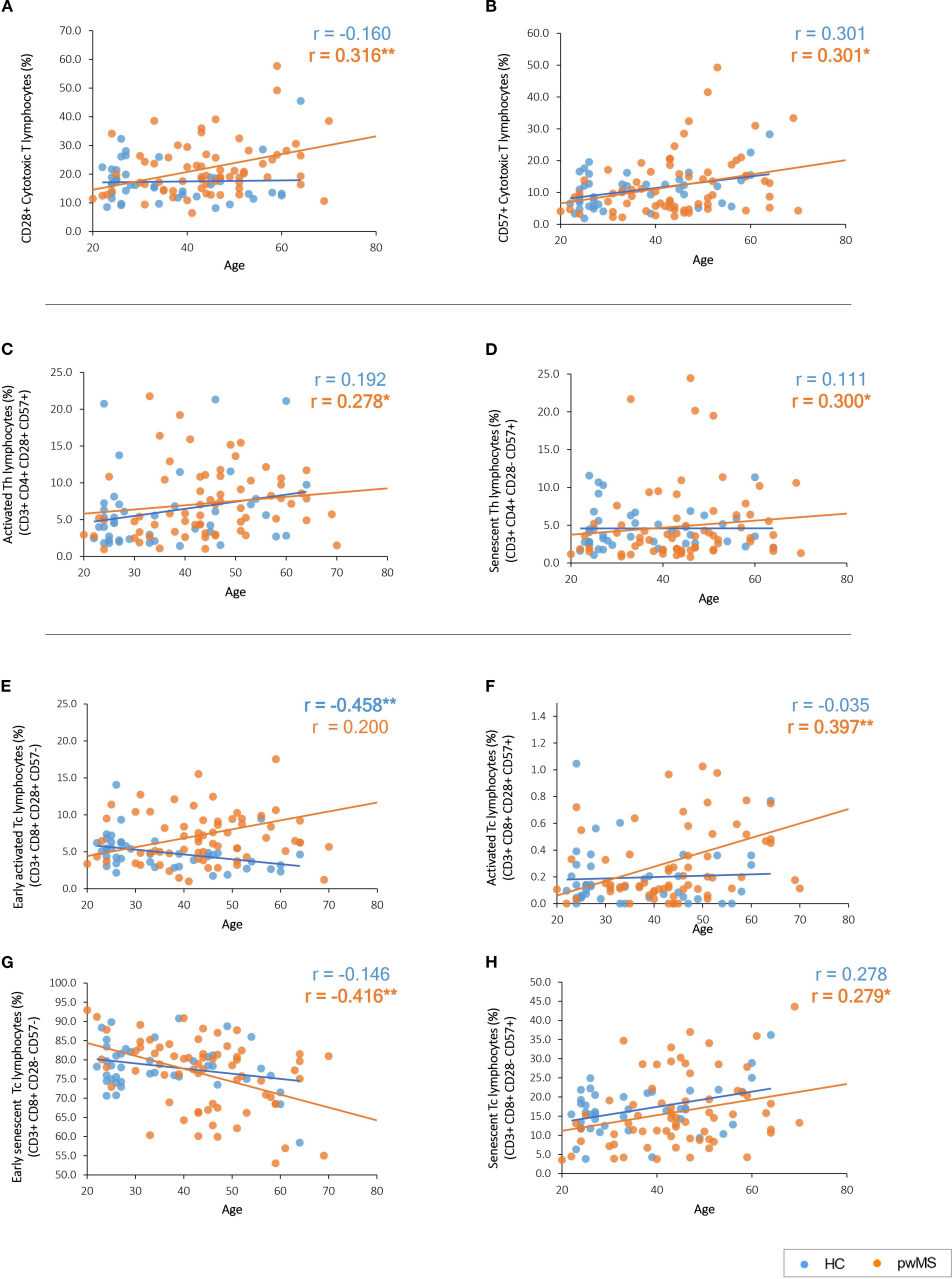
Graphical representation of the significant correlations between age and the immune subpopulations based on CD28 and CD57 expression. **(A, B)** CD28 and CD57 expression with age. **(C, D)** Age-related correlations of T helper cell subpopulations. **(E–H)** Age-related correlations of cytotoxic T cell subpopulations. Significant results are highlighted in bold.

Regarding CD28 and CD57, their expression, increases in cytotoxic T cells with age, but only in pwMS (p=0.009 and p=0.013, respectively) (**Supplementary Table S2** and **Figures 2A, B**).

Notably, the classification of the different T-cell subpopulations based on the expression of both CD28 and CD57 revealed different correlations in the study groups (**Supplementary Table S2**). PwMS presented an increase with age of activated cells (CD28+ CD57+) in Th lymphocytes (p=0.022) (**Figure 2C**) and Tc lymphocytes (p=0.001) (**Figure 2F**) subpopulations. The same pattern is observed for senescent cells (CD28- CD57+) that increased with age in pwMS both in Th lymphocytes (p=0.013) (**Figure 2D**) and Tc lymphocytes (p=0.021) (**Figure 2H**). In addition, early senescent Tc lymphocytes (CD28- CD57-) significantly decrease (p=0.001) with age in pwMS (**Figure 2G**).

No significant correlations were found for these subpopulations in HCs (**Supplementary Table S2**). However, HCs present a decline in early activated Tc lymphocytes (CD28+ CD57-) (p=0.002) with age that is not observed in pwMS (**Figure 2E**). None of the variables analyzed in the third FC panel, designed for the characterization of the immune T cell response, showed significant correlations with age (**Supplementary Table S2**).

### 3.3 Thymic involution is more pronounced in younger people with MS

To gain insight into the age-related changes observed in the immune system of pwMS, thymic involution was studied by measuring sjTREC. These DNA molecules have been widely described as biomarkers for age determination, due to their age-related decline and their association with a reduction of naïve T cells in the elderly (35, 36).

**Figure 3:**
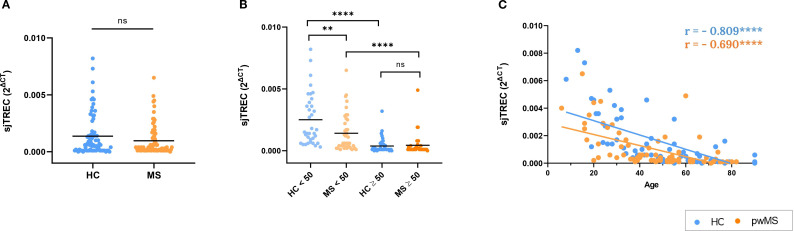
Results of the thymic involution study by the analysis of sjTREC relative amount. **(A)** Comparison between HCs and pwMS. **(B)** Analysis of sjTREC levels, using the 50-year age cutoff **(C)** Correlations of sjTREC with age. *p<0.05; **p<0.01; ***p<0.001; ****p<0.0001.

First, sjTREC levels in both groups were compared to understand the overall differences between HCs and pwMS. The results showed that the relative amount of sjTREC did not differ between pwMS and HCs (p=0.09) (**Figure 3A**). Next, sjTREC results were compared with 50 years as a cutoff point. This has been defined by our group and others as a critical age for the immune system aging (37, 38). The results of this analysis showed that in individuals under 50, pwMS have lower levels of sjTREC than those of HCs. In contrast, no differences were found in individuals over 50 years. In addition, as expected, both HCs and pwMS showed lower sjTREC levels when the over 50 years subgroup was compared to the respective under 50 years subgroup (**Figure 3B**). Lastly, correlation analysis was performed between age and the relative amount of sjTREC. The results showed a significant decrease in sjTREC levels with age for HCs (r=-0.809, p<0.0001) and pwMS (r=-0.690, p<0.0001) (**Figure 3C**).

### 3.4 Increased inflammatory markers in people with MS with age

Another key feature of immunosenescence is inflammaging, the chronic state of low-grade inflammation that is the result of an unbalanced regulation of the immune system (21, 39, 40). Here we measured characteristic markers – the proinflammatory molecules IL-1β, IL-6, IL-8, TNF-α and C-reactive protein (CRP) and the anti-inflammatory IL-10 – to evaluate inflammaging in pwMS. Note that IL-1β levels were consistently below the limit of detection of the kit in the vast majority of the study samples and, therefore, results were not included in the analysis.

**Figure 4:**
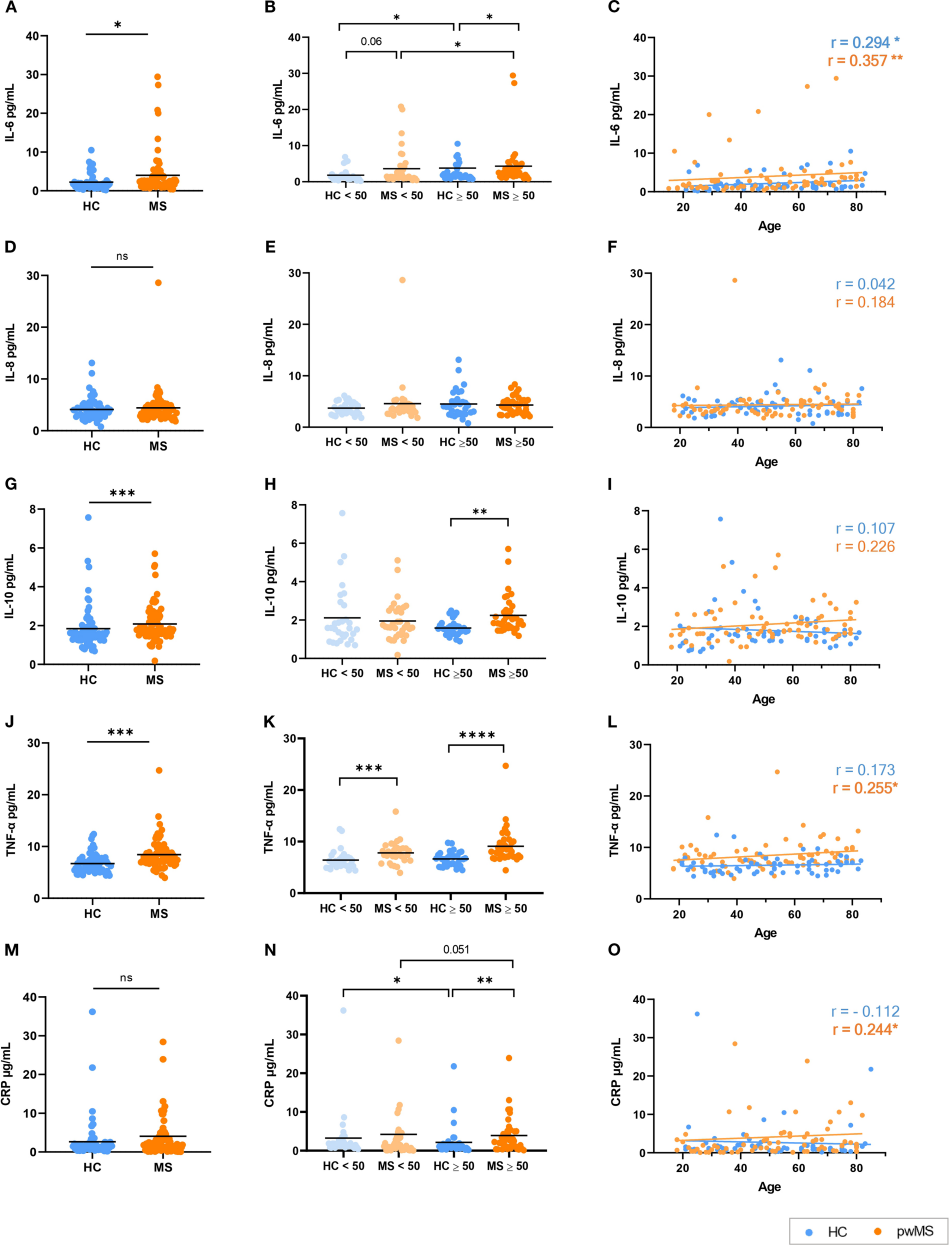
Analysis of the inflammaging markers in HCs and pwMS. **(A–C)** IL-6, **(D–F)** IL-8, **(G–I)** IL-10, **(J–L)** TNF-α and **(M–O)** CRP. *p<0.05; **p<0.01; ***p<0.001; ****p<0.0001.

People with MS showed significantly higher levels of IL-6 compared to HCs (p=0.021) (**Figure 4A**). When groups were classified in people under/over 50 years, an increase in IL-6 with age was found for both HCs and pwMS. Notably, a trend towards increased IL-6 was observed in pwMS under 50 compared to their HC counterparts (p=0.06), and this difference reached statistical significance in the over 50 group (p=0.037) (**Figure 4B**). In addition, IL-6 levels showed a significant positive correlation with age in both groups (**Figure 4C**) (r=0.357, p=0.002 for MS, r=0.294, p=0.019 for HC).

IL-8 results were analyzed following the same strategy. The comparison of IL-8 levels between HCs and pwMS revealed no significant differences between groups (**Figure 4D**). Similarly, no significant differences were found when the under/over 50 years groups were compared (**Figure 4E**), and no correlation was observed between IL-8 levels and age in either group (**Figure 4F**).

People with MS exhibited elevated levels of IL-10 when compared to HCs (p<0.001) (**Figure 4G**). Using a cutoff point of 50 years, no significant differences in IL-10 levels were observed either within groups or between HCs and pwMS in individuals under 50. However, pwMS over 50 years exhibited significantly higher IL-10 levels (p=0.001) (**Figure 4H**). No significant correlation was observed between IL-10 levels and age in either the HC or MS groups (**Figure 4I**).

In the case of TNF-α, its levels were significantly higher in pwMS than in HCs (p < 0.0001), as shown in **Figure 4J**. When comparing the under/over 50 years groups, pwMS exhibited significantly higher TNF-α levels compared to HCs in both the under 50 (p<0.001) and over 50 (p<0.0001) age groups (**Figure 4K**). Regarding correlations, a positive correlation between age and TNF-α levels was found for the MS group (r=0.255, p=0.032) but no significant correlation was observed in HCs (**Figure 4L**).

Lastly, for CRP, while no significant differences were observed in CRP levels between all pwMS and HCs (**Figure 4M**), when classified as under/over 50 years, pwMS had higher CRP levels than HCs in the over 50 years comparison. Surprisingly, for HCs, lower CRP levels were reported in individuals over 50 years when compared to the under 50 group (**Figure 4N**). A positive correlation was observed between CRP levels and age only in pwMS (r=0.240, p=0.040) (**Figure 4O**).

### 3.5 NFL is elevated even in younger people with MS

To further investigate the intricate relationship between inflammation, aging, and MS disease, we also analyzed NFL, a well-established biomarker of neurodegeneration (41).

**Figure 5:**
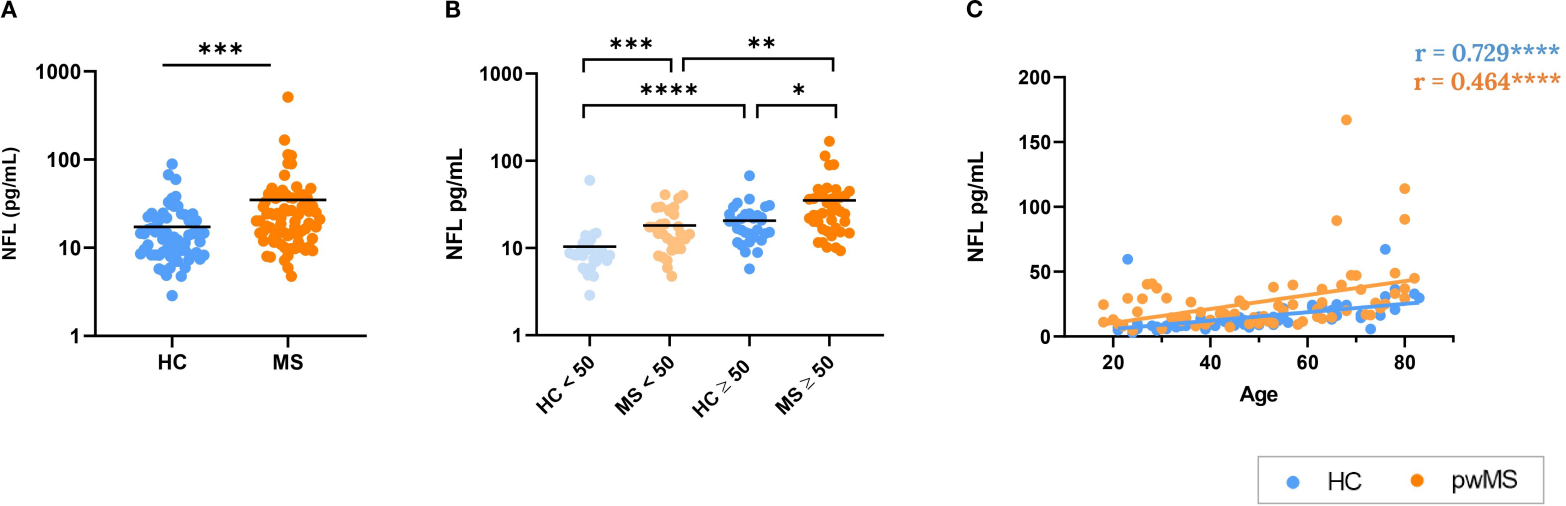
Analysis of neurofilament light (NFL) as a biomarker of neurodegeneration. **(A)** Comparison between HCs and pwMS. **(B)** Analysis of NFL levels with the 50 year cutoff. **(C)** Correlations of NFL with age. *p<0.05; **p<0.01; ***p<0.001; ****p<0.0001.

In our samples, pwMS exhibited significantly higher levels of NFL when compared to HCs (p < 0.001) (**Figure 5A**). Remarkably, pwMS exhibited significantly higher NFL levels compared to HCs in both the under 50 (p<0.001) and over 50 (p=0.046) age groups. Besides, both pwMS and HC groups presented significant differences between the under and over 50 years subgroups (**Figure 5B**). A positive correlation was observed between NFL levels and age in both pwMS and HC groups (r=0.465, p<0.0001 for MS, r=0.729, p<0.0001 for HC) (**Figure 5C**).

### 3.6 NFL correlates with inflammatory mediators only in people with MS

**Figure 6:**
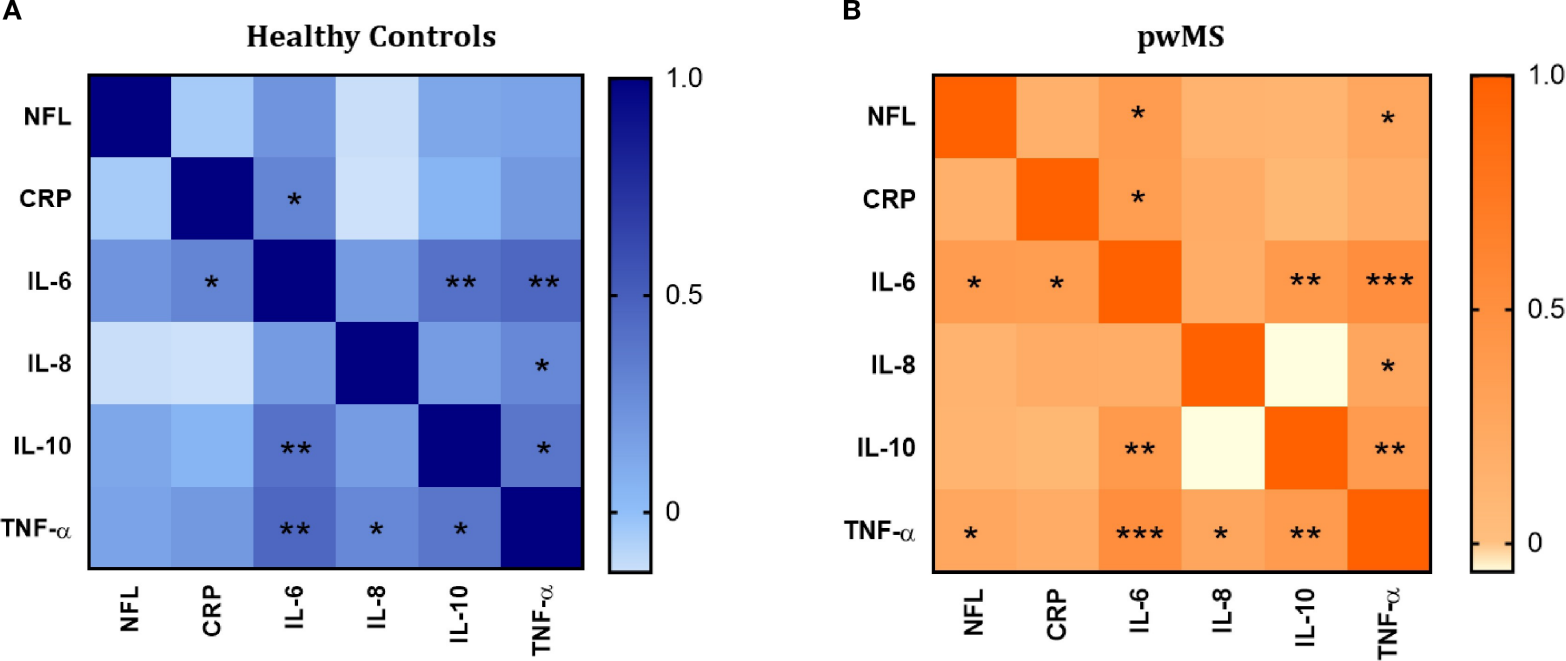
Correlation analysis between the inflammatory mediators (IL-6, IL-8, IL-10 and TNF-α and CRP) and neurofilament light (NFL). **(A)** Correlation matrix in healthy controls. **(B)** Correlation matrix in pwMS. Significant correlations are indicated with asterisks.

Since data about inflammatory markers and NFL levels were obtained in the same donors (Plasma Cohort), correlations between all the variables were evaluated. In the case of HCs (**Figure 6A**), IL-6 correlates positively with CRP (r=0.300, p=0.014), IL-10 (r=0.399, p=0.001) and TNF-α (r=0.453, p<0.001). TNF-α also correlates positively with IL-8 (r=0.287, p=0.019) and IL-10 (r=0.379, p=0.002).

The pattern of positive correlations observed in HCs was also present in the MS group. Specifically, in pwMS, IL-6 showed positive correlations with CRP (r=0.361, p=0.001), IL-10 (r=0.394, p<0.001), and TNF-α (r=0.504, p<0.0001). TNF-α correlated positively with IL-8 (r=0.256, p=0.022) and IL-10 (r=0.376, p=0.001). Additionally, in contrast to HCs, positive correlations between NFL and IL-6 (r= 0.374, p=0.001), and NFL and TNF-α (r=0.254, p=0.029) were found in pwMS.

### 3.7 Telomere attrition in multiple sclerosis mirrors that of healthy controls

**Figure 7:**
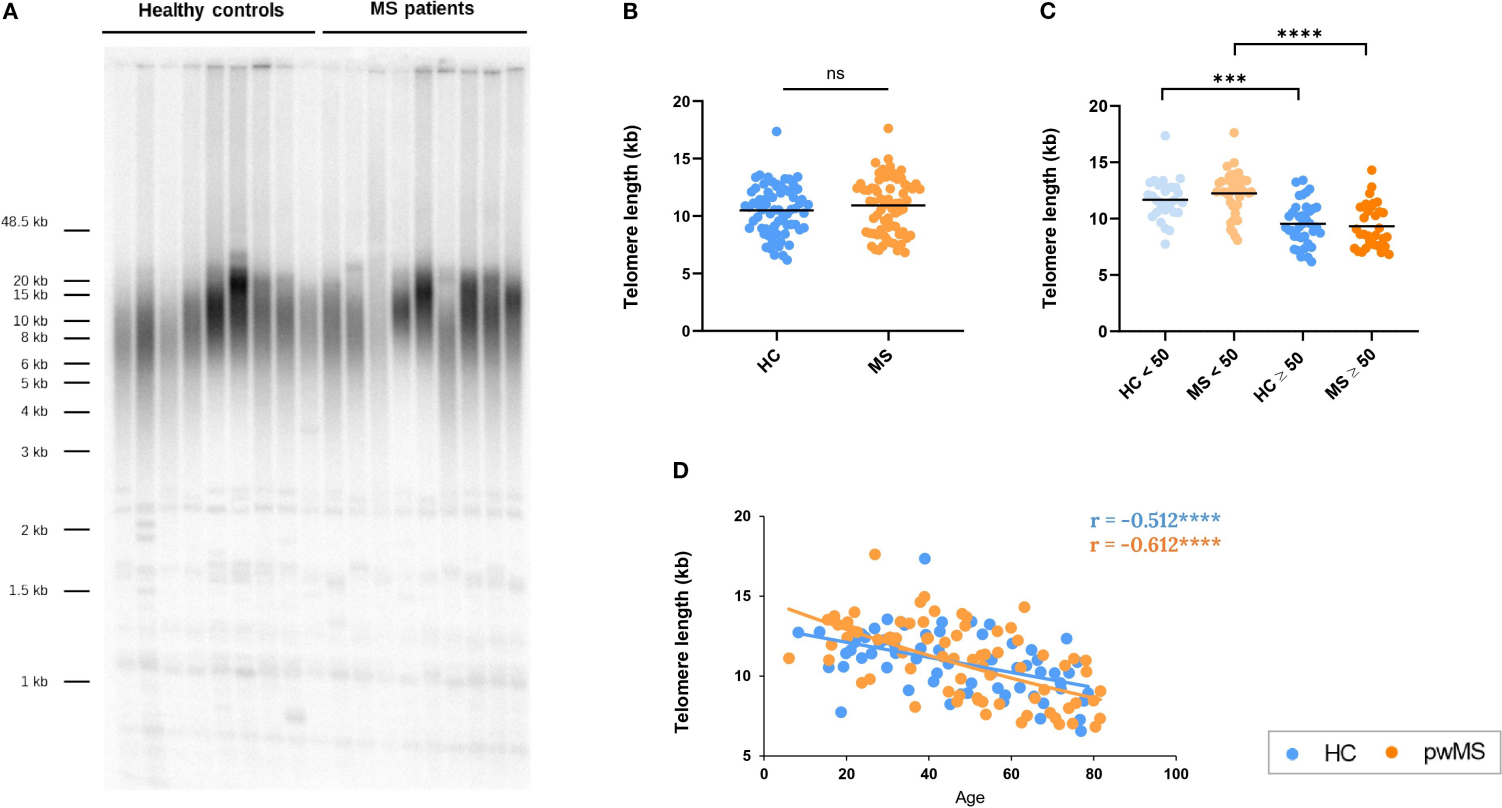
Assessment of telomere length in pwMS and HCs. **(A)** Representative image of Terminal restriction fragment (TRF) length analysis. **(B)** Comparison of the mean telomere length between HC and pwMS. **(C)** Analysis of mean telomere length, using the 50-year age cutoff. **(D)** Correlation of telomere length with age. *p<0.05; **p<0.01; ***p<0.001; ****p<0.0001.

To further explore how aging affects the immune system of pwMS, we investigated telomere attrition over time, a well-established hallmark of aging (42, 43). TRF length analysis showed no significant differences in mean telomere length between HCs and pwMS (**Figure 7B**). Similarly, no differences were found when comparing the under/over 50 years groups (**Figure 7C**). A significant age-dependent decrease in telomere length was observed in both HC (r =-0.511, p<0.0001) and MS groups (r=-0.612, p<0.0001) (**Figure 7D**). These results confirmed telomere attrition with age, and showed that progression is similar in HCs and pwMS.

Taking advantage of the fact that sjTREC and telomere length data were obtained in the same donors (DNA Cohort), correlations between the variables were evaluated. A positive correlation was observed between sjTREC levels and telomere length in both HC (r= 0.565, p<0.001) and pwMS (r= 0.530, p<0.001).

## 4 Discussion

Multiple sclerosis is a chronic inflammatory and neurodegenerative disease in which, the dysregulation of the immune system plays a fundamental role (1). Given the increasing life expectancy of pwMS (15, 44), it is essential to understand how the aging of the immune system affects the disease pathophysiology. Indeed, immunosenescence results in alterations in immune cell populations, chronic inflammation, and reduced regenerative capacity (18, 21), which could influence disease progression and response to therapies in aged pwMS. Our study aimed to characterize the immunosenescence process in pwMS, in a wide range of ages. Thus, we performed a comprehensive analysis and examined differences in immune cell populations, thymic involution, inflammatory mediators, neurodegeneration biomarkers, and telomere attrition.

The flow cytometry analysis revealed that while the main immune cell populations did not show significant differences between pwMS and HCs, some subpopulations presented variations. Notably, Th lymphocytes were significantly increased in pwMS. More specifically, activated T subsets (CD28+CD57+) were significantly increased in pwMS, while early senescent T subsets (CD28-CD57-) were decreased, which could be reflecting the heightened immune activation characteristic of the disease.

The correlation analysis of immune cell populations with age revealed distinct age-related changes between HCs and pwMS. B cell proportions increased with age in HC, whereas no correlation was found in pwMS. Conversely, NK lymphocytes showed an age-related increase only in pwMS. It has been extensively described in the literature that memory B cells increase with age, which could explain the total B cell increase with age in HC (16, 21). The fact that this increase in B cells does not occur in pwMS could be due to the role they play in the disease, which could result in an exhaustion or dysfunction of B cells. Regarding NK cells, it has been described that aging causes phenotypic and functional alterations in NK cells, including a reduction in their cytotoxic activity and cytokine production and response, although the total number of NK cells is usually maintained or slightly increased (16, 23, 45, 46). In the case of MS, the implications of NK subpopulations in the disease are being investigated but, to our knowledge, previous studies did not evaluate age-related changes in NK cells in pwMS (47, 48).

Our findings indicate that the expression of CD28 and CD57 antigens increase with age in cytotoxic T cells of pwMS. We expected the increase of CD57 as it is a known senescence marker and it is described that CD57+ cells increase with chronic immune activation as well as during human aging (32, 49, 50). On the contrary, the CD8+CD28+ age-related increase was not expected, as it is widely reported that the expression of CD28, along with the activation capacity of T cells, is lost progressively with age (32, 51–54). Consequently, these results suggest that different immunological mechanisms could be operating in MS. Our results are in line with the research of Zuroff et al. that reported that pwMS exhibited abnormal age-associated increases of activated (HLA-DR+CD38+) T cells (55). Conversely, some papers have reported same blood levels of CD8+CD28+ Tc in pwMS and HCs (56, 57). However, these works did not explore age-related correlations. We hypothesize that the observed increase in CD8+CD28+ T cells with age in MS reflects an accumulation or sustained activation of this cytotoxic subset, which contrasts with the typical age-related decline seen in healthy individuals. This persistent activation may indicate heightened immune system activity and inflammation, which are known contributors to disease progression and poorer prognosis in older pwMS (47, 58).

As for the subpopulations characterized based on CD28 and CD57 expression, we observed that pwMS present a positive correlation in activated Th cells (CD4+CD28+CD57+) and senescent Th cells (CD4+CD28-CD57+) with age. These results partially support immunosenescence in MS, as the accumulation of CD4+CD28− T cells is a main characteristic of aging (16). Furthermore, our results are in line with previous articles that described an increase in CD4+CD28- cells with age in MS that was not observed in HCs up to 60 years old (24). Regarding cytotoxic cells, our results showed a positive correlation of CD8+CD28+CD57- cells with age only in HCs. Besides, CD8+CD28+CD57+ cells demonstrated a positive correlation with age in pwMS while CD8+CD28-CD57- cells significantly decreased with age in pwMS which, to our knowledge, had not been evaluated before. These populations showed no correlations with age in HCs.

Finally, CD8+CD28-CD57+ cells showed a positive correlation with age in pwMS. In HCs, the same trend did not reach statistical significance, despite the increase of this subpopulation with age has been described in many previous studies (59–61). We believe that the unexpected results for HCs in this as well as in the other subpopulations could be due to the limited number of samples of HCs over 50 years of the *PBMCs Cohort*. Notably, a good sample size was achieved in the MS group and, to the best of our knowledge, there are no prior studies addressing these subpopulations and their age-related correlations in pwMS, so our work represents a significant contribution to the MS field.

Next, thymic involution was assessed through sjTREC quantification, a recognized biomarker of thymic function. Both pwMS and HCs showed the expected negative correlation of sjTREC with age. When comparing between the two study groups, no overall differences in sjTREC levels were observed between pwMS and HCs but, importantly, age-specific analyses revealed significant differences. In individuals under 50 years, a lower amount of sjTREC was observed in pwMS compared to HCs, indicating greater thymic involution in this age group. In contrast, in individuals over 50 years, a decline in the relative amount of sjTREC occurred in both pwMS and controls, with both groups reaching similar levels. This observation shows that while thymic involution occurs in both groups, it affects earlier to pwMS. Our results are in line with previous studies that describe early thymic involution in MS, suggesting the potential involvement of the thymus in CNS autoimmunity (24, 62–64).

With regard to the analysis of inflammatory mediators in plasma, it revealed a consistent increase of IL-6, IL-10, and TNF-α in pwMS compared to HCs. Besides, IL-6 levels correlated positively with age in both groups, and the analysis between the individuals under/over 50 years showed that older pwMS have higher IL-6 than younger pwMS. These results aligns with the concept of inflammaging and its characteristic senescence-associated secretory phenotype (SASP). IL-6 is a key soluble factor within the SASP, known to increase with age and to contribute to the inflammaging phenotype (39, 65–67). In the case of MS, the presence of IL-6 in acute and chronic active lesions of pwMS has been demonstrated (68). In addition, pwMS had higher levels of IL-6 in plasma and cerebrospinal fluid (CSF) compared to people with other neurologic diseases (69–72). To the best of our knowledge, this is the first time that the concentration of IL-6 in plasma including pwMS of all ages was evaluated, and an increase of IL-6 with age was found in pwMS.

In the case of TNF-α, it also had a positive correlation with age in pwMS and higher concentrations in pwMS under and over 50 years when compared to the respective HCs. TNF-α is another cytokine widely implicated in MS pathology and aging. Elevated TNF-α levels have been detected in active CNS lesions, as well as in the serum and CSF of pwMS (73, 74). As IL-6, TNF-α is one of the key cytokines in SASP and its levels increase with age contributing to inflammaging (67). It should be noted that, in our results, HCs did not show this increase, while pwMS exhibited a significant age-related increase in TNF-α levels. With these results, and considering that some previous works also found no differences in TNF-α with healthy aging (75), we hypothesize that the implication of TNF-α could be even more relevant in MS disease.

For IL-10, elevated levels were found in pwMS, particularly in individuals over 50 years. As IL-10 is an anti-inflammatory cytokine that leads to decreased release of TNF, IL-1β, IL-6 and IL-8 among other cytokines and it also has the capacity to act in a neuroprotective manner, its increase may represent a compensatory response to persistent immune activation (76, 77). Moreover, some studies have reported an increase in IL-10 levels with age, suggesting a compensatory mechanism to counteract the rise in pro-inflammatory cytokines associated with immunosenescence (39, 78, 79). However, the role of IL-10 in MS remains controversial. Some studies showed that IL-10 levels vary depending on the treatment received (80) and that there are fluctuations in IL-10 levels depending on disease phase, with decreases prior to a relapse and increases during remission (77). Similarly, some research suggests a potential regulatory role for IL-10 in MS (81–83), while others point towards a possible pro-inflammatory function (84, 85).

CRP, a systemic inflammation marker, did not show significant overall differences between pwMS and HCs. This finding aligns with previous literature, which also reports no significant differences in CRP levels between pwMS and HCs (86). However, pwMS over 50 years had higher CRP levels than respective HCs and a positive correlation with age, indicating an age-dependent increase in systemic inflammation in MS. Unexpectedly, older HCs showed lower CRP levels compared to younger HCs. This is in sharp contrast to most of the previous literature that reported higher CRP levels in older individuals (75, 87). Some studies suggest that older adults with a healthy lifestyle and training, have CRP levels comparable to or even lower than less healthy younger individuals (88–90), but these aspects are out of the scope of the present work.

NFL, a biomarker of neuronal damage, was elevated in pwMS compared to HCs. In addition, NFL levels correlated positively with age in both groups, reflecting the expected age-related neurodegenerative processes. These results were anticipated, as NFL has already been recognized as a reliable biomarker for MS, playing a crucial role in the preclinical phase, diagnosis, prognosis, and monitoring of both the disease and its treatments (41, 91, 92). Additionally, it has been well-documented that NFL levels increase with age, which is consistent with our findings, reflecting the neurodegenerative processes associated with aging (93). It is noteworthy, that increased NFL was already found in pwMS under 50 years, showing that neurodegeneration occurs earlier and more aggressively in MS.

When exploring the potential correlations between the inflammatory and neurodegenerative markers, our results revealed distinct correlation patterns in pwMS and HCs. In HCs, IL-6, IL-10 and TNF-α had positive correlations with each other, and IL-6 with CRP, suggesting a general inflammatory response. This pattern was also observed in the MS group. Furthermore, in pwMS, we found positive correlations between NFL and IL-6, as well as between NFL and TNF-α, which were not found in the HC group. These results suggest that, in MS, the interplay between inflammation and neurodegeneration may be more pronounced, with neuronal damage showing a stronger relationship with key inflammatory mediators like IL-6 and TNF-α. This highlights the potential role of inflammation in driving neurodegenerative processes in MS.

Finally, our study of telomere length revealed no significant differences in mean telomere length between pwMS and HCs. Both groups exhibited an expected age-associated decrease in telomere length, with a stronger correlation in pwMS. In line with our results, Hug et al. explored telomere length in T cells of MS and HCs by TRF and observed a decline of telomere length with age in subjects with MS as well as in HCs in both CD4+ and CD8 +. In addition, they did not find any statistically significant difference in telomere shortening between pwMS and HCs, mirroring our results (94).

Prior literature presented a discordant picture: some papers describing an association between longer telomere length and a higher risk of MS susceptibility (95) while others reported shorter leukocyte telomeres in pwMS (96). According to a systematic review, pwMS generally have shorter telomeres in their blood cells compared to healthy individuals, although a full consensus has not yet been reached due to diverse results (97). It should also be noted that telomere shortening is tissue-specific and that variations in research methods can contribute to the heterogeneous findings regarding telomere length in pwMS.

Our results reveal a positive correlation between sjTREC levels and telomere length in both HCs and pwMS. This correlation was biologically expected, as both biomarkers decline with age and are affected by cell division. sjTRECs are non-replicating DNA fragments that become diluted with each T-cell division (33), while telomeres shorten with each cell cycle (43). Further research is needed to determine whether these processes are biologically linked or just influenced by aging.Several limitations should be noted when interpreting the findings of this study. First, as previously indicated, the small sample size of HCs over 50 years of the *PBMCs Cohort* limit the interpretation of the age-related changes of immune populations in HCs. However, we consider that the results of pwMS, which are the main focus of the work, are reliable. Second, it should be mentioned that we designed the study with no exclusion criteria based on EDSS score, MS subtype and treatment. This decision increases the heterogeneity of the study group, but at the same type reinforces the relevance of the reported aging-associated differences despite heterogeneity.

In conclusion, our study provides a multifaceted characterization of immunosenescence in people with MS, including individuals across a broad age range. By simultaneously assessing multiple biomarkers—from immune cell subsets to inflammatory mediators and thymic output—we addressed a significant gap in the literature. Our key novel findings include distinct alterations in T cell subpopulations, with increased activated T cells in pwMS, potentially reflecting the immune activation of the disease. Age-related changes in immune cell populations differed between pwMS and HCs in key immune populations, like B and NK cells, and distinct patterns of CD28 and CD57 expression on T cells. Our novel findings also indicate earlier thymic involution in younger pwMS and an age-related increase of inflammatory markers, particularly IL-6, TNF-α and CRP in pwMS. We also found elevated NFL levels in pwMS, with a positive correlation with age observed in both groups. Notably, a unique finding was that NFL correlated positively with IL-6 and TNF-α only in pwMS. In addition, telomere attrition increased with age in both pwMS and HCs.

These results underscore the complex interplay between aging and immune dysregulation in MS and highlight the need for further research to fully elucidate the mechanisms driving these age-related immune changes and their impact on MS disease. Moreover, it emphasizes the importance of taking age into account for the prognosis and treatment of the disease.

## Data Availability

The raw data supporting the conclusions of this article will be made available by the authors, without undue reservation.
