# Supplementary material for: Aging-dependent immunological changes in multiple sclerosis
Source: Front Immunol. 2025 Oct 2;16:1663526. doi: 10.3389/fimmu.2025.1663526 (PMC12529361; doi:10.3389/fimmu.2025.1663526)
Supplement: Supplementary file 1 [file DataSheet1.pdf]

Supplementary Table 1. Age distribution across the different cohorts included in the study.

Cohort	Status	Age (years old)							
		<20 (F/M)	20-29 (F/M)	30-39 (F/M)	40-49 (F/M)	50-59 (F/M)	60-69 (F/M)	70-79 (F/M)	≥80 (F/M)
PBMCs Cohort (n=110)	HC (n=42)	-	21 (16/5)	7 (6/1)	7 (6/1)	4 (4/-)	3 (2/1)	-	-
	pwMS (n=68)	1 (1/-)	5 (5/-)	15 (11/4)	24 (17/7)	15 (12/3)	6 (4/2)	1 (1/-)	1 (1/-)
DNA Cohort (n=150)	HC (n=75)	5 (2/3)	10 (5/5)	11 (7/4)	9 (5/4)	10 (7/3)	10 (5/5)	10 (6/4)	10 (7/3)
	pwMS (n=75)	6 (2/4)	9 (6/3)	11 (5/6)	14 (8/6)	12 (6/6)	10 (5/5)	9 (6/3)	4 (4/-)
Plasma Cohort (n=142)	HC (n=66)	-	10 (5/5)	10 (5/5)	10 (5/5)	10 (5/5)	10 (5/5)	10 (5/5)	6 (3/3)
	pwMS (n=76)	7 (3/4)	10 (5/5)	10 (5/5)	11 (5/6)	11 (6/5)	12 (6/6)	10 (6/4)	5 (5/-)

Supplementary Table 2. Age-Related Correlations of Immune Cell Populations in Multiple Sclerosis and Healthy Controls. Note that significant correlations were marked in bold.

Immune cell populations	Healthy Controls		People with MS	
	R	p-value	R	p-value
**Main immune populations**				
**Lymphocytes**				
T lymphocytes CD3+	0,09	0,59	-0,13	0,28
**B lymphocytes CD19+**	**0,33**	**0,03**	0,14	0,26
**Natural Killer cells CD56+**	0,30	0,06	**0,38**	**0,00**
**Monocytes**				
Classical monocytes CD14++ CD16-	-0,29	0,06	0,24	0,05
Intermediate monocytes CD14+ CD16+	0,27	0,09	-0,22	0,07
**Non-classical monocytes CD14+ CD16++**	**-0,33**	**0,03**	0,06	0,65
**Aging markers and T cells subsets**				
CD28+ cells				
CD28+ T lymphocytes CD3+ CD28+	-0,06	0,68	0,02	0,85
CD28+ Th lymphocytes CD3+ CD4+ CD28+	0,02	0,90	-0,04	0,76
**CD28+ Tc lymphocytes CD3+ CD8+ CD28+**	-0,16	0,31	**0,32**	**0,01**
CD57+ cells				
CD57+ T lymphocytes CD3+ CD57+	0,24	0,12	0,20	0,11
CD57+ Th lymphocytes CD3+ CD4+ CD57+	0,24	0,12	0,20	0,10
**CD57+ Tc lymphocytes CD3+ CD8+ CD57+**	0,30	0,05	**0,30**	**0,01**
T lymphocytes				
Early activated T lymphocytes CD3+ CD28+ CD57-	-0,06	0,69	-0,07	0,56
**Activated T lymphocytes CD3+ CD28+ CD57+**	0,19	0,24	**0,27**	**0,03**
Early senescent T lymphocytes CD3+ CD28- CD57-	-0,05	0,76	-0,21	0,09
Senescent T lymphocytes CD3+ CD28- CD57+	0,10	0,55	0,23	0,06
T helper lymphocytes CD3+ CD4+	0,26	0,10	-0,03	0,84
Early activated Th lymphocytes CD3+ CD4+ CD28+ CD57-	-0,07	0,64	-0,16	0,20
**Activated Th lymphocytes CD3+ CD4+ CD28+ CD57+**	0,20	0,21	**0,28**	**0,02**
Early senescent Th lymphocytes CD3+ CD4+ CD28- CD57-	0,01	0,97	-0,05	0,70
**Senescent Th lymphocytes CD3+ CD4+ CD28- CD57+**	0,11	0,48	**0,30**	**0,01**
T cytotoxic lymphocytes CD3+ CD8+	-0,07	0,65	-0,09	0,45
**Early activated Tc lymphocytes CD3+ CD8+ CD28+ CD57-**	**-0,46**	**0,00**	0,20	0,10
**Activated Tc lymphocytes CD3+ CD8+ CD28+ CD57+**	-0,03	0,83	**0,40**	**0,00**
**Early senescent Tc lymphocytes CD3+ CD8+ CD28- CD57-**	-0,15	0,36	**-0,42**	**0,00**
**Senescent Tc lymphocytes CD3+ CD8+ CD28- CD57+**	0,28	0,07	**0,28**	**0,02**
**T cell response**				
Active T helper lymphocytes CD3+ CD4+ CD69+	0,03	0,84	-0,04	0,75
Th17 lymphocytes CD3+ CD4+ CD196+	-0,09	0,58	-0,16	0,21
T regulatory lymphocytes CD3+ CD4+ CD25+ CD127-	-0,02	0,90	-0,13	0,31
